# Rediscovery of *Haematobosca zuluensis* (Zumpt), (Diptera, Stomoxyinae): Re-description and amended keys for the genus

**DOI:** 10.1186/1756-3305-5-267

**Published:** 2012-11-21

**Authors:** Leo Braack, Adrian C Pont

**Affiliations:** 1 Zoonoses Research Unit, Department of Medical Virology, Faculty of Health Sciences, University of Pretoria, Pretoria, South Africa; 2 Oxford University Museum of Natural History, Parks Road, Oxford, OX1 3PW, U.K

**Keywords:** Haematobosca, Biting flies, Stomoxyinae

## Abstract

**Background:**

Prior to this publication, the biting fly *Haematobosca zuluensis* (Zumpt, 1950) (Diptera, Muscidae, Stomoxyinae) was known only from a single male specimen collected in 1923 in Zululand, South Africa. Seven additional males were subsequently captured in the Kruger National Park of South Africa, one in 1984 and six in 1991, but remained unidentified until now. The genus includes species of considerable veterinary significance, but current keys for identification of species are misleading due to inadequate description of *H. zuluensis*.

**Methods:**

External morphological features are described to enable species characterization, including intraspecific variability.

**Results:**

This paper confirms the existence of *H. zuluensis*, expands its known range, provides a full description of males of the species, and gives an up to date set of keys for the 15 known species within the genus. Available records suggest that *Haematobosca zuluensis* is a low density species as yet known only from wildlife areas of South Africa.

**Conclusions:**

The additional specimens of *H. zuluensis* have enabled an improved description of the species and an improved set of keys to identify constituent members of the genus.

## Background

Based on a single male specimen caught by R H Harris in February 1923 in northern KwaZulu-Natal Province (previously known as ‘Zululand’), South Africa, Zumpt [1] initially described the fly as *Haematobia zuluensis*, but in his 1973 monograph placed it in the genus *Haematobosca*. No other specimens of the species have been recorded since 1923, until this publication. Taxonomic treatment of the genus *Haematobosca* has remained remarkably stable since the 1973 monograph by Zumpt, with only two new species described since that time, *H.croceicornis*[2] from Gabon, and *H. aurata*[3] from Kenya; *H. alcis* has also been recognized as a good Holarctic species [4].

One of us (Braack) in January 1984 collected one male *Haematobosca* near Tshalungwa Spring, and in March 1991 six more males close to another nearby freshwater spring, Magovani, both sites located in the northern region of the Kruger National Park, South Africa. Initially these flies were considered a new species because of perceived differences with the written description of *H. zuluensis* by Zumpt [5], but they were not described and published in the hope of finding females for a comprehensive description. Despite fairly intensive collections of biting flies as part of routine parasitological work in Kruger National Park over two decades, no additional specimens of this species were captured. Recent re-examination of the Kruger Park specimens and comparison with the type specimen of *H. zuluensis,* suggests that they belong to the same species; earlier doubts had been based on intraspecific variation in some features used in the 1973 key provided by Zumpt.

Pont & Dsouli [2] developed a new set of keys for the separation of *Haematobosca* species, but without having access to the *H. zuluensis* type specimen the keys were misleading and *H. zuluensis* was not keyed out correctly.

Because of the intraspecific variation in some morphological features used in the keys of Zumpt [5] and Pont & Dsouli [2], the species is re-described below and a revised set of keys provided.

## Methods

Pinned, dry specimens of the seven flies collected in Kruger National Park, and the holotype specimen, all examined under stereoscopic microscope, formed the basis of the descriptions below.

## Results

### Re-description: *Haematobosca zuluensis* (Zumpt, 1950)

#### Male

##### Head

Eyes large, red, with small uniformly-sized facets across entire area bearing no setulae/microtrichiae; hind margin of eye slightly concave in the lower half; large median ocellus and two smaller posterior ocelli, all red (except in specimens initially kept in ethanol, following which ocelli turn opaque); frons black; parafacial dark orange; fronto-orbital plate as well as parafacial and facial ridge with shiny silver pruinosity when viewed with a particular angle of light; antenna bright yellow-orange, except for basal one-third of arista which is slightly darkened in some specimens (but still essentially orange); gena black but suffused with an underlying dark orange hue which becomes more dominant in the vibrissal area; oral margin orange; palpus orange with multiple stout, dark setulae distally; proboscis orange but with blackened tip.

##### Thorax

Ground colour of scutum black but overlaid with a silvery-grey pruinosity; two sets of prominent black vittae on the presutural part of scutum, one being a pair of narrow medial lines traversing the entire length of the presutural area (and continuing postsuturally but narrowing to become indistinct when reaching scutellum), each of these medial lines adjoined laterally by a slightly thicker short oblique line which commences midway along the presutural scutum and curves inwards towards the medial pair of vittae, tapering rapidly and fading away before reaching the suture; in the postsutural area, midway between the two medial lines, a single thick black vitta stretching over the distal half but fading away before reaching the scutellum; anterior spiracle orange; posterior spiracle appearing black but the adjoining sclerites suffused with an underlying dark orange hue; proepisternal depression bare; postpronotal lobe covered medially and ventrally with approximately 30 moderate-sized setae, but three prominent setae dorsally; several stout proepisternal setae (sometimes obscured by attached phoretic mites); katepisternum with 2 stout setae, one anterior and one posterior, and a liberal sprinkling of finer setae along the hind margin; proepimeral seta present; meron bare; notopleural bristles 1:1.

##### Wing

Clear, with no infuscated areas; basicosta and tegula orange; anterior costal margin fringed with setulae along entire length; vein R_1_ bare except in one specimen (Tshalungwa) which has a single dorsal setula close to the junction with costa; vein R_1_ with no setae ventrally; vein R_2+3_ without setulae; vein R_4+5_ dorsally with three or four widely separated setulae before junction with cross-vein r-m (but completely absent in three specimens); cell r_4+5_ at wing-tip wider than length of cross-vein r-m; vein M curved forward towards vein R _4+5_ in terminal part; cross-vein dm-cu clearly concave; both calypters pale orange; haltere orange.

##### Legs

All segments of all legs yellow-orange, covered with black setae, except for apical tarsomeres of fore and hind legs which are slightly darkened in some specimens; fore tibia without submedian seta, but with two stout setae posteriorly at distal tip; mid-tibia with one posterior seta sub-medially, located between the two rows of finer setulae running lengthways down ventral tibia; mid-femur with two or three stout setae posteriorly close to distal tip; hind femur with one stout anteroventral seta a short distance from junction with tibia; hind tibia usually with one stout seta ventrally at distal end; hind tibia usually with one posterior submedian seta (present in five, absent in two); in at least some specimens, hind tibia also with one long ventral hair clearly longer and thinner than any of the other tibial setae.

##### Abdomen

Ground colour grey/black but suffused by a dark orange hue in the anterior two tergites (in some specimens all tergites with dark orange hue).

##### Measurements

**Figure 1:**
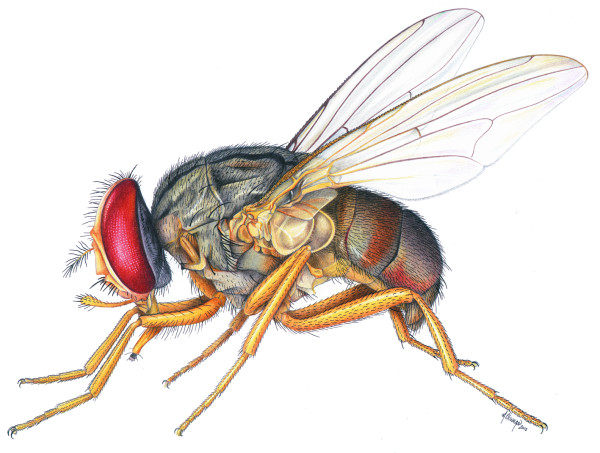
**Lateral view of *Haematobosca zuluensis *(Zumpt).**

(based on 5 specimens): Width of head 1.88mm - 2.10mm (viewed dorsally, outer edge of one eye to outer edge of the other eye); depth of eye 0.58mm - 0.88mm (viewed dorsally, anterior to posterior margins in region of greatest depth); length of postpedicel 0.38mm – 0.48mm; width of thorax in postsutural area 1.90mm - 2.38mm (dorsal view); length of thorax from anterior margin to distal tip of scutellum 2.88mm – 2.96mm; width of abdomen in area of greatest extent 2.25mm – 2.89mm (viewed dorsally); length of abdomen 2.38mm - 2.85mm (dorsal view) Figure 1.

## Discussion

*Haematobosca zuluensis* is one of only two species (the other being *Haematobosca croceicornis* Pont & Dsouli, 2008) of the genus which have an entirely yellow/orange antennal postpedicel (3rd antennal segment); it differs from *H. croceicornis* as follows:

a. *zuluensis* has the terminal (apical) half of the arista yellow, whereas the terminal half is dark in *croceicornis*;

b. in *zuluensis* the lateral areas of the scutum and also the pleura are dark, as opposed to yellow in *croceicornis*;

c. in *zuluensis* vein R_1_ dorsally has at most one setula apically, whereas *croceicornis* usually has several setulae (although in some specimens it remains bare);

d. in *zuluensis* vein R_4+5_ has no setulosity in the dorsal apical area, which is setulose in *croceicornis*;

e. *zuluensis* is recorded from South Africa, geographically far removed from the known distribution of *croceicornis* in Gabon.

### Intra-specific variation

There is definite variation between specimens ― clearly belonging to the same species ― in the morphological traits used by Zumpt [5] and Pont & Dsouli [2] for keying out individuals of *Haematobosca* to species level, resulting in those keys being of limited use, at least for identifying *H. zuluensis*. As an example, one of us (Braack) examined a specimen of *H. squalida* (Natal Museum, South Africa) identified by Zumpt himself, which according to his 1973 keys should have 3–5 ventral hairs on the hind tibia, but they are absent in this particular specimen which nevertheless by all other features qualifies as a true *H. squalida*. From the description of *H. croceicornis* by Pont & Dsouli (2008), it is clear that considerable variation occurs in the setulosity on the branches of the radial veins.

In the seven male *Haematobosca* captured from two sites closely located to each other in Kruger National Park, the specimens obviously belong to the same species, but nevertheless differ in the presence and arrangement of setulae on the wings and legs, which affects the ability to key out these individuals using existing keys (the variation in number of setulae is not caused by secondary loss of such structures, as there are no empty sockets indicating that setulae had been present earlier). These features are detailed below:

i. Wing-vein R_1_: The Tshalungwa Spring specimen has one setula distally on the dorsal length of the vein, whereas none of the six Magovani specimens have any setulosity on R_1_. The 1923 holotype specimen has been damaged over the years: the anterior portions of both wings are completely broken away and lost (as also one mid-leg completely broken away). The key provided by Zumpt [5] suggests that distally vein R_1_ is bare in *H. zuluensis*, thus corresponding to the Magovani specimens.

ii. Wing-vein R_4+5_: The Tshalungwa specimen and two of the five Magovani flies have no dorsal setulae along the entire length of the vein; in the other three flies there are three or four clear setulae dorsally in the proximal part of the vein before crossvein r-m; vein R_4+5_ in the 1923 holotype is described in Zumpt [5] as being the *‘…same as in H. squalida*’, thus implying it to be *‘…partly setulose*’, again in agreement with at least some of the Magovani specimens.

iii. In one of the Magovani flies there appears to be no submedian posterior seta on the mid-tibia , but it is present in the others;

iv. In two of the Magovani flies, there is no submedian posterior seta on the hind tibia, present in the other five;

v. Most importantly in terms of ability to use either the Zumpt [5] or Pont & Dsouli [2] keys, only one fly (from Magovani) has a long ventral hair on the hind tibia; none of the other six have such a hair. In the specimen which does have the hair, it differs clearly from the submedian setae elsewhere on the hind tibia, by being thinner and longer, and is located anterodorsally two-thirds down the length of the tibial shaft. This hair is clearly visible in the holotype.

vi. In the holotype, and two of the Magovani specimens, the basal part of the arista is as yellow/orange as all the other segments of the antenna, whereas five other specimens have the basal part of the arista slightly darkened.

vii. Zumpt [5] states that the abdomen of the holotype specimen is broader than long; re-examination of the holotype reveals that the particular specimen has the abdomen in-curved ventrally, making it very difficult to measure; this brings some doubt to the interpretation by Zumpt that the abdomen is broader than long. In the Kruger National Park specimens, those measured had the abdomen slightly longer than broad.

Useful to note is the consistency of two features across all specimens, one being that the postpedicel is clearly and completely yellow-orange throughout its length, and the other is that the posterior crossvein (dm-cu) is clearly incurved.

Any doubts regarding possible differences between the Zululand holotype and the Kruger National Park specimens should be considered within the context of the variation between specimens caught even at the same site (Magovani Spring), the intraspecific variation observed within other species of the genus, and most importantly the relative geographic proximity of Zululand and northern Kruger National Park. *Haematobosca zuluensis* appears to be a rare, low-density species, probably encouraging some degree of genetic drift.

### Holotype

The 1923 holotype male is deposited in the insect collection of the Biosystematics Division of the Plant Protection Research Institute, Agricultural Research Council, Pretoria. Anterior portions of both wings fairly extensively damaged and one mid-leg lost.

### New material

One male, Tshalungwa Spring (S22^o^3240; E31^o^0150), Kruger National Park, South Africa, caught (Braack) with hand net 26 January 1984, plus one male Magovani Spring (S22^o^3537; E31^o^0043), Kruger National Park, South Africa captured (Braack) 5 March 1991 using Malaise trap with 70% ethanol as collecting medium, subsequently pinned and dried; in collection of Biosystematics Division, Plant Protection Research Institute, Agricultural Research Council, Pretoria. Five male specimens, captured (Braack) Magovani Spring (S22^o^3537; E31^o^0043), Kruger National Park, South Africa, ) 5 March 1991 using Malaise trap with 70% ethanol as collecting medium, subsequently pinned and dried ; in Insect Collection of Kruger National Park Research Department, Skukuza, Kruger National Park, South Africa.

## Conclusions

The additional specimens of *H. zuluensis* have enabled an improved description of the species and an improved set of keys to identify constituent members of the genus, as set out below:

### Key to the species of *Haematobosca* Bezzi (adapted from Zumpt [5], and Pont & Dsouli [2])

#### Males

1. Wing with a well-demarcated blackish-brown patch in subcostal wing cell. Thorax and abdomen black, with olive-brown dust, the dark pattern faintly developed. Fore-margin of wing infuscated. Vein R_1_ apically and vein R_4+5_ for almost entire length with setulae. Legs mainly yellow-brown. Hind tibia without ventral setae or setulae. Antennal postpedicel pale at base. 6.0 - 7.0 mm. Afrotropical………………….***wooffi*** (Zumpt, 1969)

– Wing with the subcostal cell no darker than the rest of the wing………………………………………2

2. Vein R_1_ densely setulose in apical half of dorsal surface. Thorax and abdomen yellow to orange, with yellow dust. Wing clear. Vein R_4+5_ setulose along its entire length. Hind tibia without ventral setae or setulae. 6.0 – 9.0 mm. Afrotropical………………….….***praedatrix*** (Enderlein, 1928)

– Vein R_1_ with at most a few small setulae on apical part of dorsal surface………………………………3

3. Meron and most of proepisternal depression setulose. Thorax and abdomen grey and olive-green dusted, the former with distinct pattern. Veins R_1_ and R_4+5_ bare. Legs black, with knees more or less broadly yellow. Hind tibia with series of long ventral setulae. 3.5 – 5.0 mm. Palaearctic……………….***atripalpis*** (Bezzi, 1895)

– Meron and proepisternal depression bare……4

4. Antennal postpedicel wholly bright yellow/orange……… ………………………………………………….5

– Antennal postpedicel black, or at most yellow-orange at base………………………………6

5. Vein R_1_ with at most one setula apically. Vein R_4+5_ dorsally without setulosity in the apical area, and at most 3 to 5 setulae in the proximal area before the junction with crossvein r-m (but sometimes none). Distal half of arista yellow. Base colour of lateral areas of scutum and also pleura predominantly grey-black, not yellow. Southern Africa……….***zuluensis*** (Zumpt, 1950)

– Vein R_1_ and also vein R_4+5_ for much of their length with setulae on their dorsal surfaces. Distal half of arista dark. Lateral areas of scutum and also pleura yellow. West Africa (Gabon) ………………***croceicornis*** Pont & Dsouli, 2008

6. Hind tibia with 3 - 5 long setulae on ventral surface. Abdomen about as long as broad. Thorax variable in colour, from rusty to yellow-brown with a dark median vitta to mainly darkened. Abdomen yellow-brown to almost wholly black. Dusting greyish-olive. Vein R_1_ bare, vein R_4+5_ partly setulose. Legs yellow. Afrotropical…………………………… ………….***squalida*** (Grűnberg, 1913)

– Hind tibia without long setulae ventrally……… …………………………………….7

7. Mid tibia with one or more posterior setae and hind tibia with an anterodorsal seta……….….8

– Mid tibia without posterior setae and hind tibia without anterodorsal seta………………………….11

8. Scutum with striking golden dust on anterior half. Vein R_4+5_ bare on dorsal surface. Femora mainly yellow but brown at least on apical part. Scutum with a distinct pattern. Wing clear. Vein R_1_ bare. 6.0 mm. Afrotropical (Kenya)………………………….… …………….***aurata*** Pont & Mihok, 2000

– Scutum with only yellowish- to brownish-grey dust on anterior part. Vein R_4+5_ with a few setulae on dorsal surface…………………………………….9

9. Legs uniformly yellow. Similar to *H.squalida*, but the grey-olive dusting of the body dense and thick. No distinct pattern visible on scutum or abdomen, the latter longer than broad. 6.0 mm. Afrotropical…… ……………………….***kangwagye***i (Zumpt, 1967)

– Legs blackish, only the knees more or less extensively yellow…………………………10

10. Abdomen with the paired lateral spots on tergites 3 – 5 large and black. Scutum with the dark vittae broad, the area of dusting very reduced. 16 – 20 pairs of shorter weaker frontal setae and setulae. Mouth-edge in profile reaching well in front of profrons. Scutum brownish-grey dusted, abdomen yellow-grey to grey, both distinctly patterned. Wing clear, vein R_1_ usually and vein R_4+5_ always with setulae basally. 5.0 – 7.0 mm. Palaearctic and Northern Oriental (Himalaya)………………**s*timulans*** (Meigen, 1824)

– Abdomen with the paired lateral spots on tergites 3 – 5 small, brown, or even absent. Scutum with the dark vittae narrow, the area of dusting very extensive. 8 – 11 pairs of longer frontal setae and setulae. Mouth edge in profile hardly in front of profrons. Scutum with lighter, yellowish-grey dusting, abdomen yellowish-grey. Otherwise as in *H. stimulans*. 4.5 – 5.5 mm. Holarctic ……… …………………………….***alcis*** (Snow, 1891)

11. Species of the Oriental region and some Pacific islands. Hind femur without a preapical anteroventral seta. Scutum and abdomen with yellowish and olive-brown dusting, the pattern similar to that of *H. stimulans*. Wing hyaline or brownish tinged, vein R_1_ bare, vein R_4+5_ with a few setulae at base. Legs varying in colour from mainly blackish to wholly yellow. 3.5 – 6.0 mm. Oriental and Pacific………… …………………….***sanguinolenta*** (Austen, 1909)

– Species of the Afrotropical region. Hind femur with a preapical anteroventral seta…………………12

12. Prosternum setulose. Palpi strongly dilated at apex… …………………………………………….13

– Prosternum bare. Palpi less strongly dilated at apex……………………………………14

13. Frontal vitta at middle narrower than the width of one fronto-orbital plate. Scutum and abdomen with mainly grey, partly yellowish dusting, scutal pattern distinct, abdominal pattern ill-defined. Wing clear, veins R_1_ and R_4+5_ bare. Legs black, knees yellow. 3.5 – 5.0 mm. … ……………………….***uniseriata*** (Malloch, 1932)

– Frontal vitta at middle about twice as wide as one fronto-orbital plate. Otherwise like *H. uniseriata*… …………………….***angustifrons*** (Malloch, 1932)

14. Fronto-orbital and scutal setulae of normal length. Dusting and pattern of scutum and abdomen as in *H. uniseriata*. Wing hyaline, veins R_1_ and R_4+5_ bare. Legs black, knees yellow. 3.5 – 5.0 mm ………………… ………………………….***latifrons*** (Malloch, 1932)

– Fronto-orbital and scutal setulae strikingly long and mostly as long as aristal hairs. Scutum almost completely black and without a distinct pattern. Abdomen with grey and olive-brown dusting, the latter forming ill-defined spots. Wing and legs as in *H. latifrons*. 5.0 mm.…………………………… …………………….***hirtifrons*** (Malloch, 1932)

#### Females [not known for *hirtifrons* (Malloch) and *zuluensis* (Zumpt)]

1. Wing with a well-demarcated blackish-brown patch in subcostal wing cell. Afrotropical ……………… …………………………….***wooffi*** (Zumpt, 1969)

– Wing with the subcostal cell no darker than the rest of the wing ………………………………………….2

2. Vein R_1_ densely setulose in apical half …………… …………….……….***praedatrix*** (Enderlein, 1928)

– Vein R_1_ with at most a few setulae on apical part of dorsal surface …………………………….3

3. Meron and most of proepisternal depression setulose. Palaearctic …….***atripalpis*** (Bezzi, 1895)

– Meron and proepisternal depression bare …….4

4. Species of the Holarctic and Oriental-Australasian regions ……………………………………….5

– Species of the Afrotropical region …………….7

5. Mid tibia without posterior setae and hind tibia without anterodorsal seta. Hind femur without preapical anteroventral setae. Oriental and Pacific ……………….….***sanguinolenta*** (Austen, 1909)

– Mid tibia with one or more posterior setae and hind tibia with an anterodorsal seta. Hind femur with 1 – 2 preapical anteroventral setae ……….6

6. Mid femur mainly yellow. Mouth-edge in profile projecting well beyond level of profrons. Dark spots on tergite 5 large. Palaearctic ………………… ……………………………….***stimulans*** (Meigen, 1824)

– Mid femur mainly black. Mouth-edge in profile hardly projecting beyond level of profrons. Dark spots on tergite 5 small or absent. Holarctic …… ……………………………***alcis*** (Snow, 1891)

7. Antennal postpedicel entirely bright yellow ……………………***croceicornis*** Pont & Dsouli, 2008 (and the as yet undiscovered female of zuluensis may also share this feature, as in the male)

– Antennal postpedicel black, or at most yellow at base ……………………………………….8

8. Legs mainly to wholly yellow …………………….9

– Legs blackish, only knees more or less extensively yellow …………………………………….11

9. Legs with the femora darkened in apical quarter or less …………………***aurata*** Pont & Mihok, 2000

– Legs wholly yellow ………………………….10

10. Antennal postpedicel 3 times as long as pedicel ……………………….***squalida*** (Grűnberg, 1913)

– Antennal postpedicel 2.5 times as long as pedicel ……………………***kangwagyei*** (Zumpt, 1967)

11. Prosternum bare. Palpi less strongly dilated at apex …………………….….***latifrons*** (Malloch, 1932)

– Prosternum setulose. Palpi strongly dilated at apex ……………………………………………12

12. Frontal vitta everywhere narrower than a fronto-orbital plate ………….***uniseriata*** (Malloch, 1932)

– Frontal vitta everywhere broader than a fronto-orbital plate…….….***angustifrons*** (Malloch, 1932)

## Competing interests

The authors declare that they have no competing interests.

## Authors’ contributions

LB conceived the need for the paper, undertook the microscopic and descriptive work as well as wrote the initial draft. ACP conducted the literature search, provided expert taxonomic editing and tracked down the location of the holotype. Both authors read and approved the final version of the manuscript.
